# Clinicopathological differences between Bartonella and other bacterial endocarditis-related glomerulonephritis – our experience and a pooled analysis

**DOI:** 10.3389/fneph.2023.1322741

**Published:** 2024-01-15

**Authors:** Mineaki Kitamura, Alana Dasgupta, Jonathan Henricks, Samir V. Parikh, Tibor Nadasdy, Edward Clark, Jose A. Bazan, Anjali A. Satoskar

**Affiliations:** ^1^ Department of Pathology, Division of Renal and Transplant Pathology, The Ohio State University Wexner Medical Center, Columbus, OH, United States; ^2^ Department of Nephrology, Nagasaki University Graduate School of Biomedical Sciences, Nagasaki, Japan; ^3^ Department of Internal Medicine, The Ohio State University Wexner Medical Center, Columbus, OH, United States; ^4^ Department of Internal Medicine, Division of Nephrology, The Ohio State University Wexner Medical Center, Columbus, OH, United States; ^5^ Department of Internal Medicine, St. Vincent Hospital, Erie, PA, United States; ^6^ Department of Internal Medicine, Division of Infectious Disease, The Ohio State University Wexner Medical Center, Columbus, OH, United States

**Keywords:** Bartonella, endocarditis, glomerulonephritis, Staphylococcus aureus associated glomerulonephritis, pooled analysis, cat-scratch disease, ANCA, proteinase-3

## Abstract

**Background:**

Although *Staphylococcus aureus* is the leading cause of acute infective endocarditis (IE) in adults, *Bartonella* spp. has concomitantly emerged as the leading cause of “blood culture-negative IE” (BCNE). Pre-disposing factors, clinical presentation and kidney biopsy findings in Bartonella IE-associated glomerulonephritis (GN) show subtle differences and some unique features relative to other bacterial infection-related GNs. We highlight these features along with key diagnostic clues and management approach in Bartonella IE-associated GN.

**Methods:**

We conducted a pooled analysis of 89 cases of Bartonella IE-associated GN (54 published case reports and case series; 18 published conference abstracts identified using an English literature search of several commonly used literature search modalities); and four unpublished cases from our institution.

**Results:**

*Bartonella henselae* and *Bartonella quintana* are the most commonly implicated species causing IE in humans. Subacute presentation, affecting damaged native and/or prosthetic heart valves, high titer anti-neutrophil cytoplasmic antibodies (ANCA), mainly proteinase-3 (PR-3) specificity, fastidious nature and lack of positive blood cultures of these Gram-negative bacilli, a higher frequency of focal glomerular crescents compared to other bacterial infection-related GNs are some of the salient features of Bartonella IE-associated GN. C3-dominant, but frequent C1q and IgM immunofluorescence staining is seen on biopsy. A “full-house” immunofluorescence staining pattern is also described but can be seen in IE –associated GN due to other bacteria as well. Non-specific generalized symptoms, cytopenia, heart failure and other organ damage due to embolic phenomena are the highlights on clinical presentation needing a multi-disciplinary approach for management. Awareness of the updated modified Duke criteria for IE, a high index of suspicion for underlying infection despite negative microbiologic cultures, history of exposure to animals, particularly infected cats, and use of send-out serologic tests for *Bartonella* spp. early in the course of management can help in early diagnosis and initiation of appropriate treatment.

**Conclusion:**

Diagnosis of IE-associated GN can be challenging particularly with BCNE. The number of Bartonella IE-associated GN cases in a single institution tends to be less than IE due to gram positive cocci, however Bartonella is currently the leading cause of BCNE. We provide a much-needed discussion on this topic.

## 1 Introduction

Post-streptococcal acute glomerulonephritis (PSAGN) has been a prototype of infection-related glomerulonephritis (IRGN) for over a century, largely prevalent in the pediatric population (1, 2) but adults are also affected. It was seen in epidemic outbreaks in the early era and continues to be endemic worldwide but more so in lower socio-economic strata and Aboriginal communities (1). In the late 1990s, cases of acute methicillin-resistant Staphylococcus aureus (MRSA) infection-associated acute glomerulonephritis (SAGN) were increasingly reported mainly in the Western subcontinents (3) and Japan (4), affecting predominantly the elderly with co-morbid conditions and also prevalent among young adults with a history of injection drug use (IDU) (2, 5). This is different from the subacute infections with Staphylococcus epidermidis in shunt nephritis or subacute endocarditis (1). Acute MRSA infections have emerged as the leading cause of IRGN in the elderly as reported in several case series (6–8) and are associated with a wide spectrum of sites ranging from superficial skin infection, cellulitis, osteomyelitis, septic arthritis, deep visceral abscesses, pneumonia, infected prosthetic devices, indwelling central catheters and infective endocarditis (IE) (2, 4–8).

Despite a large focus on PSAGN and SAGN, cases of Bartonella infective endocarditis (IE)-associated GN are also increasingly encountered and reported time and again, but only as case reports or small case series since not too many cases are seen in any single institution. The largest single-center cohort of Bartonella IE-associated GN was described by Boils et al. comprising of 4 patients out of a total of 49 patients with IE (5). The French registry has described a total of 106 cases of Bartonella IE diagnosed over a period of 9 years (from 2005 to 2013), but IE-associated GN was not studied in that series (9). Staphylococcus species is reported as the leading cause of acute IE in adults, implicated in up to 53% of the cases according to Boils et al. (5), Bartonella has also emerged as one of the leading causes of culture-negative IE among gram-negative bacilli (9, 10), frequently associated with destructive valvular lesions, and concomitant high titer anti-neutrophil cytoplasmic antibodies (ANCA) with proteinase-3 (PR-3) specificity, posing an even bigger diagnostic challenge (11–13). More so, clinical presentations can vary widely and can affect both pediatric and older populations. The predisposing factors and histopathology of the immune complex GN associated with Bartonella IE can show unique features and subtle differences from other causes of IRGN. We aimed to compile the published reports on Bartonella IE-associated GN and describe the four cases from our institution to highlight the peculiarities and subtle clinico-pathologic differences from other bacterial IE-associated GN. A workflow for management is also provided.

## 2 Manuscript formatting

### 2.1 Methods

#### 2.1.1 Pooled analysis and controls

For the pooled analysis, we did an English literature search using PubMed, Scopus, Embase, Science Direct, ProQuest, Springer, Wiley online library, and Google Scholar with the search words “Bartonella”, “endocarditis”, “glomerulonephritis” and “biopsy”. We identified a total of 984 possible published case reports, and conference abstracts over a period of 22 years, ranging from 2001 to 2022. We also searched for abstracts presented on “Bartonella” at the American Society of Nephrology-kidney week from 2003 to 2022 (available online), and 20 abstracts were identified. The diagnosis of IE was based on the modified Duke criteria (13, 14), and cases with at least “possible” IE defined by the Duke criteria were included. In short, the modified Duke criteria consists of two major and five minor clinical criteria. Major: 1. Blood culture positive for typical microorganisms for IE, and 2. evidence of endocardial involvement. Minor criteria: 1. predisposing heart condition, or injection drug use, 2. fever (>38°C), 3. vascular phenomena, such as major arterial emboli, conjunctival hemorrhages, Janeway lesions 4. immunologic phenomena, such as glomerulonephritis, 5. Microbiological evidence (serological evidence of active infection with organisms). For diagnosis of “possible” IE, one major criterion and one minor criterion, or three minor criteria are required to be fulfilled. Janeway’s lesions are included in vascular phenomena, but Osler’s node and Roth’s spots are classified in immunologic phenomena (13). Cases with “fever” or “intermittent fever”, except “low grade fever”, were regarded as having fever (>38°C).

**Figure 1:**
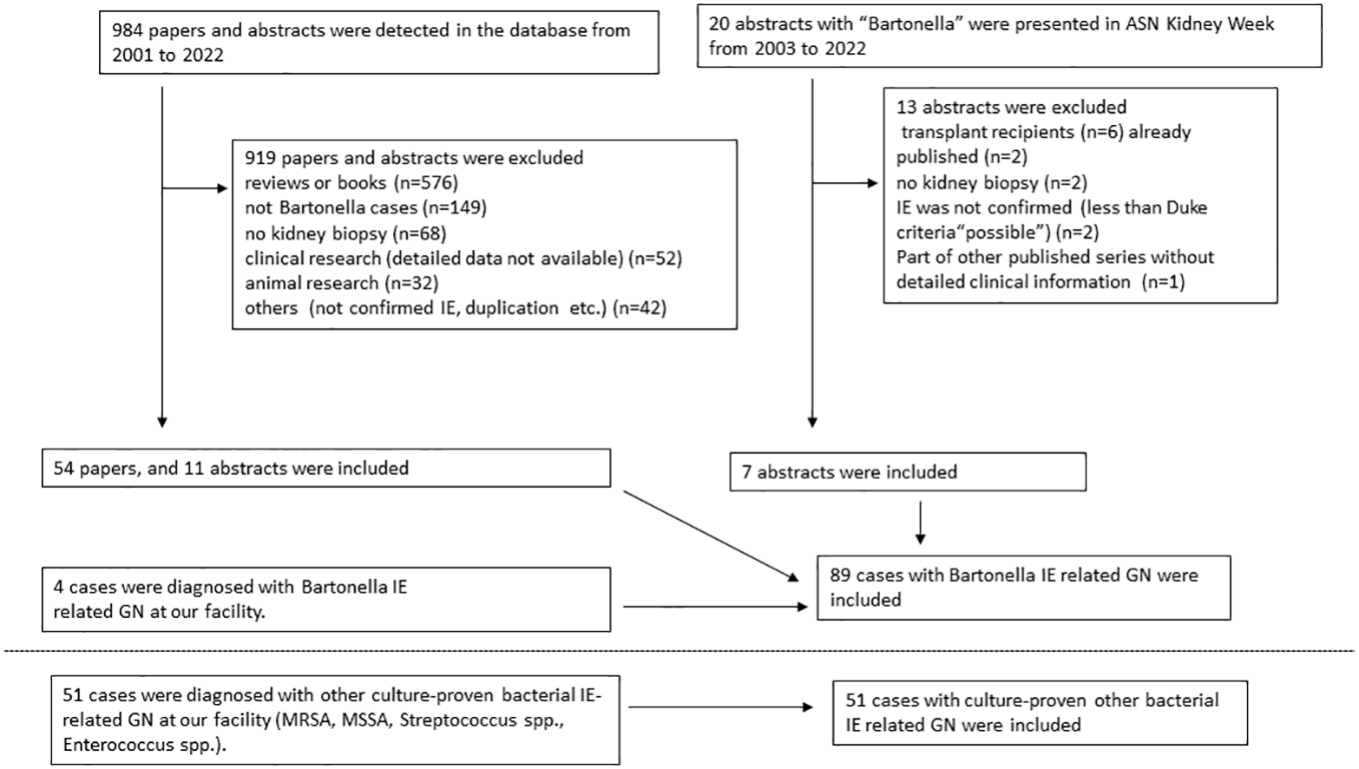
Flowchart depicting inclusion criteria for pooled analysis of published reports on Bartonella endocarditis related glomerulonephritis.

Exclusion criteria for cases in this study were as follows: cases reports on animals, literature without definitive evidence of Bartonella IE or lacking sufficient details on individual cases, literature on kidney transplant recipients, and conference abstracts from the same cases already published as case reports. Selection of cases for the pooled analysis is shown in the flow diagram (**Figure 1**). Also, data from Boils’s series (5) on IE-associated GN (which contained 4 cases of Bartonella IE) is shown.

For the four previously unpublished cases of Bartonella IE-associated GN from our institution, we present detailed clinico-pathologic data. For comparison, we have also present data from the native kidney biopsies of culture-proven IE-related GN caused by bacteria other than Bartonella, that were diagnosed at the Ohio State University Wexner Medical Center from 2004 to 2022 (7, 15).

#### 2.1.2 Data curation

Diagnostic biopsy evaluation at our institution was done as described previously (7, 15). The time from onset of symptoms to the biopsy was estimated from the descriptions provided in the published manuscripts or from the pathology reports (for cases from our institution). If reported cases indicated “hypercellularity” in glomeruli without specifying, mesangial or endocapillary, we regarded the cases as having both mesangial and endocapillary hypercellularity. We considered “any crescents” (we did not separate between cellular, fibrocellular and fibrous crescents) because of limited details provided in many of the published reports. Cases with necrotizing lesions in glomeruli were considered to have cellular crescents because the past study showed that necrotizing areas were not observed in cases without crescents (5). The intensity of immunofluorescence staining was graded (0-3) at our facility. Similarly, this rule was applied to reported cases if the intensity of immunofluorescence was shown with grade. Evaluation of renal outcome after treatment was performed only in cases with Bartonella IE-associated GN, based on the published reports and from our hospital records for the four cases from our institution. For the non-Bartonella IE cases, follow-up information for renal outcome is not shown because more than half of the biopsies were received from outside referring hospitals and detailed follow-up data is not easily available.

#### 2.1.3 Ethics

This study was approved by the Ohio State University Internal Review Board (IRB 2011H0364, 2022H0005) and was conducted under the Declaration of Helsinki.

#### 2.1.4 Statistical analyses

Numerical data was shown with mean ± standard deviation or median with (interquartile range). Categorial data was shown with number (%). Numerical and categorical data were compared using the Wilcoxon signed-rank test and chi-square tests, respectively. All statistical analyses were conducted using the JMP Pro 15. P<0.05 was considered statistically significant.

### 2.2 Results

#### 2.2.1 Patient background and characteristics

A total of 89 cases with Bartonella IE-associated GN were included in this study (54 published case reports and case series (16–69), and 18 (17 published, 1 presented) conference abstracts (70–87), and four cases diagnosed at our institution). A flowchart for including cases is shown in **Figure 1**. In addition, 51 cases with culture-proven IE-related GN due to other microorganisms from our institution were included for comparison. The summary of cases with Bartonella IE-associated GN and IE-related GN caused by other microorganisms is shown in **Tables 1**–**3**. Along with that, the data from the case series of Boils et al (5) of IE-related GN is also shown. The detailed descriptions of the four cases of Bartonella IE-associated GN from our institution are summarized in **Tables 4**, **5** (depicted as Cases 1-4).

**Table 1:** Demographic features of cases with endocarditis-associated glomerulonephritis caused by Bartonella and other microorganisms.

	IE due to Bartonella (previous reports) (N=89)	IE due to other microorganisms (OSU cohort) (N=51)	P value	IE related GN cases from Boils et al., 2015 (N=49) (4 cases were Bartonella+)
**Age (years)**	52 ± 19 (n=87)	47 ± 15 (n=51)	0.051	48 (n=49)
**Age range**	(6−79)	(19−75)		(3−84)
**Male: Female**	75:12 (n=87)	36:15 (n=51)	0.03	38:11 (n=49)
**Estimated time to biopsy** **(days)**	90 (30-150) (n=67)	30 (30-83) (n=40)	<0.001	N/A
**ANCA+**	52, (75%) (n=69)	7, (29%) (n=24)	<0.001	8, (28%) (n=29)
**PR3; CANCA**	35; 5	5; 0		Not available
**MPO; pANCA**	2; 2	1; 1		Not available
**Both PR3 and MPO**	5	0		Not available
**Atypical**	3	0		Not available
**ANA+**	17, (31%) (n=54)	3, (10%) (n=30)	0.02	4, (15%) (n=26)
**Cryoglobulin+**	13, (39%) (n=33)	2, (14%) (n=14)	0.08	N/A
**Rheumatoid factor+**	26, (84%) (n=31)	11, (52%) (n=21)	0.01	N/A
**Serum creatinine (mg/dL)**	3.7 ± 1.6 (n=74)	4.9 ± 2.7 (n=49)	0.02	3.8 (1.0-12) (n=37)
**Low C3 (yes)**	47, (78%) (n=60)	19, (49%) (n=39)	0.002	17, (53%) (n=32)
**Low C4 (yes)**	35, (58%) (n=60)	14, (36%) (n=39)	0.03	6, (19%) (n=32)
**Proteinuria (yes)**	62, (98%) (n=63)	37, (97%) (n=38)	0.72	1.8 g/day (n=18)
**Hematuria (yes)**	62, (97%) (n=64)	43, (98%) (n=44)	0.79	36, (97%) (n=37)

Numerical data is shown in mean with standard deviation or median with interquartile range.

Categorial data is shown with number with percentage.

Wilcox ranked sum test and chi-square test are used to compare two groups.

ANCA, anti-neutrophil cytoplasm antibodies; ANA, antinuclear antibodies, IE, infective endocarditis; N/A, not available.

**Table 2:** The details of endocarditis (confirmation, microorganisms, location, specific patient background, and associated symptoms).

	IE due to Bartonella (N=89)	IE due to other microorganisms (N=51)	Boils et al., 2015 (N=49) (4 cases Bartonella+)
Blood culture			
**positive**	4, (4%) (n=89)	51, (100%) (n=51)	47, (96%) (n=49)
**negative**	85, (96%) (n=89)	0, (0%) (n=51)	1, (2%) (n=49)
**unknown**	0, (0%) (n=89)	0, (0%) (n=51)	1, (2%) (n=49)
Confirmation of microorganisms (multiple methods were done)			
**Blood culture**	4, (4%) (n=89)	51, (100%) (n=51)	47, (96%) (n=49)
**Serology (blood)**	80, (99%) (n=81)	N/A	1, (2%) (n=49)
**Serology or culture (tissue)**	4, (27%) (n=15)	All Culture blood and/or tissue	Not available
**PCR (blood)**	23, (72%) (n=32)	N/A	Not available
**PCR (tissue)**	31, (86%) (n=36)	N/A	Not available
**Others (Western blotting, immunohistochemically, Warthin-Starry stain etc.),**	16 cases	N/A	Not available
**Unknown**	0, (0%) (n=89)	N/A	Not available
Microorganisms			
B. henselae*	69, (78%) (n=89)	N/A	4, (8%) (n=49)
B. quintana*	22, (24%) (n=89)	N/A	0
**B. spp**	11, (12%) (n=89)	N/A	0
**Other Bartonella**	1, (1%) (n=89)	N/A	0
**MRSA**	N/A	25, (49%) (n=51)	14, (29%) (n=49)
**MSSA**	N/A	14, (27%) (n=51)	9, (18%) (n=49)
**Staphylococcus (not classified)**	N/A		2, (4%) (n=49)
**Streptococcus**	N/A	4, (8%) (n=51)	11, (22%) (n=49)
**Others**	N/A	8, (16%) (n=51)	4, (8%) (n=49)
Location of endocarditis			
**Aortic valve**	51, (65%) (n=78)	7, (19%) (n=37)	12, (29%) (n=42)
**Mitral valve**	24, (31%) (n=78)	9, (24%) (n=37)	14, (33%) (n=42)
**Tricuspid valve**	10, (13%) (n=78)	25, (68%) (n=37)	18, (43%) (n=42)
**Pulmonic valve**	7, (9%) (n=78)	0, (0%) (n=37)	2, (5%) (n=42)
**(Multiple locations involved)**	16, (21%) (n=78)	3, (8%) (n=37)	5, (12%) (n=42)
**Other**	3, (4%) (n=78)	0, (0%) (n=37)	1, (2%) (n=42)
**Unknown or not available**	11, (12%)	14, (27%)	7, (14%)
Duke criteria (confirmed with available data)			
**Definite**	74, (83%)	51, 100%	Not available
**>Possible**	15, (17%)	0, 0%	Not available
**Histories of contact with animals**	50 cases (56%) had animal contact histories.	N/A	N/A
Most plausible causes of endocarditis			
**Intravenous drug abuse**	0, (0%)	31, (61%)	N/A
**Skin infections**	0, (0%)	2, (4%)	N/A
**Hygiene problems**	1, (1%)	3, (6%)	N/A
**Cat**	42, (46%)	0, (0%)	N/A
**Dog**	2, (2%)	0, (0%)	N/A
**Multiple animals (including cat)**	6, (7%)	0, (0%)	N/A
**Others**	0, (0%)	4, (8%)	N/A
**Unknown, N/A**	39, (44%)	11, (22%)	N/A
**Prehistory of valve replacement, cardiac valvular disease, intracardiac shunt, congenital anomalies**	61, (69%)	8, (16%)	15, (30%)
**History of intravenous drug use**	4, (4%); also had cats	31, (61%)	14, (29%)
Vascular phenomena: major arterial emboli, septic pulmonary infarcts, mycotic aneurysm, intracranial hemorrhage, conjunctival hemorrhage**	at least 27 cases had vascular phenomena	at least 20 cases had vascular phenomena	N/A
Skin lesions associated endocarditis** (rash, purpura, erythematosus)	at least 29 cases had skin lesions	at least 9 cases had skin lesions	N/A

Numerical data is shown in mean with standard deviation or median with interquartile range.

Categorial data is shown with number with percentage.

PCR, polymerase chain reaction; MRSA, methicillin-resistant Staphylococcus aureus; MSSA, methicillin- susceptible Staphylococcus aureus; N/A, not available; IE, infective endocarditis.

*Sixteen cases had both B. henselae, and B. quintana infection.

** As the clinical information is limited, more cases might have had vascular phenomena and skin lesions.

Microorganisms “Others” include Enterococcus fecalis, Acinetobacter, Anerococcus prevotii, Enterobacter and methicillin sensitive Staphylococcus epidermidis.

**Table 3:** Pathological features of cases with endocarditis associated glomerulonephritis caused by Bartonella and other microorganisms.

	IE due to Bartonella (N=89)	IE due to other microorganisms (N=51)	P value	Boils et al., 2015 (N=49) (4 cases Bartonella+)
Light microscopy				
**M-hypercellularity present**	43, 91% (n=47)	43, 84% (n=51)	0.27	6, 10% (n=49)
**E-hypercellularity present**	40, 85% (n=47)	35, 69% (n=51)	0.052	27, 43%
**Any crescent present (cellular and/or fibrocellular)**	59, 83% (n=71)	31, 61% (n=51)	**0.006**	26, 53% (n=49)
**Necrotizing lesions present**	32, 68% (n=47)	22, 43% (n=51)	**0.02**	22, 45% (n=49)
Immunofluorescence*	positive or ≥1+ (0-3)	≥1+ (0-3)		≥1+ (0-3)
**IgG positive on glomeruli** **(excluding trace cases)**	24, 46% (n=52)	25, 49% (n=51)	0.77	13, 27% (n=49)
**IgA positive on glomeruli** **(excluding trace cases)**	19, 35% (n=54)	37, 73% (n=51)	**<0.001**	14, 29% (n=49)
**IgM positive on glomeruli** **(excluding trace cases)**	41, 75% (n=55)	26, 51% (n=51)	**0.01**	18, 37% (n=49)
**C3 positive on glomeruli** **(excluding trace cases)**	53, 90% (n=59)	47, 92% (n=51)	0.67	46, 94% (n=49)
**C1q positive on glomeruli** **(excluding trace cases)**	33, 69% (n=48)	12, 24% (n=51)	**<0.001**	N/A
Electron microscopy				
**Only mesangial EDD present**	27, 87% (n=31)	44, 88% (n=50)	0.90	87%
**Mesangial and capillary wall EDD present**	23, 74% (n=31)	40, 80% (n=50)	0.54	47%

Numerical data is shown in mean with standard deviation or median with interquartile range.

Categorial data is shown with number with percentage.

Wilcox ranked sum test and chi-square test are used to compare two groups.

M, mesangial; E, endocapillary; Ig, immunoglobulin; EDD, electron dense deposits; IE, infective endocarditis; N/A, not available.

*type of crescents is only available in 32 cases with Bartonellosis.

Bold values indicate significant P-values.

**Table 4:** Clinical and serologic features of the 4 in-house cases of Bartonella IE-associated glomerulonephritis.

	Case 1	Case 2	Case 3	Case 4
**Demographic features**	68 Caucasian male	79 Caucasian male	64 Caucasian male	74 Caucasian male
**Past history of valvular disease/surgery**	MVR, AVR (remote Strep anginosus endocarditis)	Bioprosthetic AVR	Thoracic aortic aneurysm repair	Bioprosthetic aortic valve
**Clinical presentation**	wt loss, fever, pancytopenia, splenomegaly	Fatigue, SOB, fluid overload, pro-BNP 34,500 pg/mL	6 months fevers, weight loss, night sweats, malaise, pancytopenia	Feeling poorly, weight loss, splenomegaly, night sweats, afebrile
**Contact with animals**	Yes (cat)	Yes (35 farm cats)	not documented	not documented
**WBC count (K/uL)**	2.58	7.3	5.0	6.2
**Hb (g/dL)**	8	7.8	9.1	not available
**Platelets (K/uL)**	97	114	68	not available
**Bone marrow examination**	Hypercellular, no dysplasia	No biopsy	Normocellular	Normocellular
**Antibiotic regimen**	Doxycycline, Rifampicin	Doxycycline, Rifampicin	IV methylprednisone,followed by oral prednisone, mycophenolate	Doxycycline, Rifampicin 2 weeks, Doxycycline alone 5 months
**Surgical treatment for endocarditis**	No	No	No (no active endocarditis)	Aortic and mitral valve replacement for disease relapse
**Proteinuria**	1.8 g/24h	1.21 g/g	2.987 g/24h	1 g/g
**Hematuria (RBC/hpf)**	>20	>20	>20	20-40
**Creatinine (0.7 - 1.3 mg/dL)**	3.75	4.2 requiring RRT	6.13	2.5
**eGFR**	17	14	10	
**Serum albumin (3.5 - 5 g/dL)**	2.4	2.7	3.2	2.4
**C3 (87-200 mg/dL)**	75	69	99	75
**C4 (18-52 mg/dL)**	14	17	23	4.8
**ANA**	1:80, speckled	Negative	Positive	Negative
**double stranded DNA**	Negative	Negative	Negative	Negative
**ANCA (Myeloperoxidase)**	Negative	pANCA positive before admission	Positive	Negative
**ANCA (Proteinase-3)**	Negative	cANCA positive before admission	Positive	weakly positive
**Rheumatoid factor**	Positive: 59	Negative	Negative	Positive
**Other positive serologies**	RNP, Ehrlichia IgG >1:2048	Absent	Absent	Absent
**Serum monoclonal protein**	IgG lambda clone	IgG lambda spike 20.8 mg/dL	IgG kappa and IgG lambda	IgG lambda
**Blood cultures**	Negative	Negative	Negative	Negative
**Bartonella henselae IgG; IgM**	1:1024; >1:20	1:4096; <1:20	1:32768; <1:20 (positive since 3 months)	>1:1024; 1:80
**Bartonella Quintana IgG; IgM**	IgG 1:512; < 1:20	Negative	IgG 1:1024; IgM < 1:20 (since 3 months)	Negative
**Bartonella PCR on the serum**	Positive	Not tested	Negative	Negative (positive on excised valve)
**Modified Duke criteria 2023 (14)**	Definite (1 major, also 4 minor)	Possible (I major 2 minor)	Definite (1 major, 3 minor)	Definite (1 major, 3 minor)
**Transesophageal echo findings**	1 cm mass on AV (TTE negative)	small fibrinous structure on AV	No vegetations	diffuse thickening of leaflet tips on the bio-prosthetic valve
**Follow-up Interval**	2 weeks	1 month	7 months	13 months
**Serum Creatinine**	4.62 mg/dL	2.46 mg/dL	3.06 mg/dL	2 mg/dL
**Hematuria; Proteinuria**	>20; 100 mg/dL (spot)	unknown	Trace; 312 mg/g (UPCR)	Negative; 600 mg/24h

**Table 5:** Biopsy features in the 4 in-house cases of Bartonella IE-associated glomerulonephritis.

	Case 1	Case 2	Case 3	Case 4
**Glomeruli**	11	22	35	22
**Globally sclerotic**	1	3	15	5
**Crescents**	1 (fibrinoid nercrosis)	0	2 cellular, 6 fibrocellular	5 (fibrinoid necrosis)
**Glomeruli with fibrinoid necrosis**	present	absent	present	present
**Glomerular exudative lesions**	absent	absent	absent	absent
**IgG**	1+ m, g	2 to3+ m	3+ m	1+ m
**IgA**	trace	2 to 3+ m	3+ m	1+ m
**IgM**	3+ m, g	3+ m	3+ m, g	0
**C3**	3+ m, g	3+ m	3+ m, g	3+ m
**C1q**	1+ m, g	3+ m	2 to 3+ m, g	0
**kappa**	2+ m, g	3+ m	3+ m, g	2+ m
**lambda**	2+ m, g	3+ m	2+ m, g	3+ m
**IFTA (interstitial fibrosis and tubular atrophy)**	mild	mild	mod	mild
**Mesangial hypercellularity**	mild	mild diffuse	mild segmental	mild segmental
**Endocapillary hypercellularity**	present, segmental	present mild (MPGN)	absent	absent
**ATN (acute tubular necrosis)**	present	present	present	present
**Electron dense deposits**	mesangial and subendothelial	mesangial and subendothelial	mesangial	Mesangial

Our pooled analysis shows that clinical presentations in patients with Bartonella IE-associated GN varied widely and usually had subacute onset, ranging from non-specific complaints such as general malaise, fatigue, weight loss, lower extremity edema, dry cough, dyspnea on exertion, arthralgias, night sweats and flu-like symptoms to more specific findings related to isolated end-organ damage such as sudden abdominal pain, mental confusion, temporal discomfort, altered mental status with syncope, acute vision loss, and cardiogenic shock. Constellation of findings including confusion, loss of balance, partial loss of vision, fatigue, weight loss, purpuric rash were present in one patient, and myalgias, fever, weight less, night sweat, and headaches in another. Most patients presented with history of fever (55.7%, confirmed or subjective), acute kidney injury (77.0%), hematuria (72.1%), and proteinuria (68.9%). A purpuric rash (31%) or swelling of the lower extremities (13.1%) were also noted. Over one third of patients (39.3%) presented with a cardiac murmur. However, given the high percentage of patients with prosthetic cardiac valves in this population, the clinical significance of this finding is uncertain. Based on the results of this pooled analysis, more than two third of Bartonella IE cases (n=61, 69%) have pre-existing heart anomaly (such as bicuspid aortic valve) or valve replacement history. Only 16% of patients with IE caused by other organisms had a history of prior valvular abnormalities.

Among the four in-house cases of Bartonella IE-associated GN, three patients had pancytopenia, splenomegaly, night sweats, weight loss, prompting a bone marrow biopsy to look for hematologic disease. All four cases had history of prior valvular repair and bioprosthetic valves. In Case 1, the valvular repair surgery was due to another prior episode of endocarditis due to *Streptococcus anginosus*. Case 3 already had history of positive *Bartonella* titers 3 months before the kidney biopsy and then developed gradual worsening in renal function while on a 3-month course of doxycycline, with development of active urine sediment, proteinuria and a biclonal spike in the serum. Also, the patient had a solitary kidney (prior nephrectomy for renal cancer), therefore kidney biopsy was initially avoided, but was subsequently performed because of rapidly worsening renal function.

Approximately half of the cases with Bartonella IE had a history of exposure to cats (n=42, 48%) or multiple animals including cats (n=6, 7%). As noted in some of the published case report, animal exposure histories from the patients may not be readily elicited. Interestingly, Case 2 from our in-house Bartonella IE cases (**Table 4**), the history of exposure to cats was discovered only after the kidney biopsy diagnosis. In fact, the patient had exposure to a large number of cats (as many as 35 in the setting of a farm). This patient presented with features of heart failure and failure to thrive. Cardiovascular disease or malignancy were the main initial clinical considerations, while underlying infection was least suspected. A purpuric rash was present in 28/89 (31%) cases of Bartonella IE-associated GN cases and 10/51 (19.6%) other IE-associated GN (**Table 2**). Interestingly, IgG lambda monoclonal proteins were detected in the serum of all our four patients (**Table 4**). One of them also had a biclonal spike with concomitant IgG kappa monoclonal protein as well. Of the 85 published cases for Bartonella IE-associated GN, 59 contained extensive laboratory information, and serum electrophoresis results were provided for 11 of these cases. Of these, only one case reported two monoclonal spikes in the serum, IgM kappa and IgM lambda, which were attributed to the patient’s cryoglobulinemia. The clinical significance of these monoclonal proteins remains uncertain. Although the possibility of a separate disease process, such as a plasma cell dyscrasia emerges, none of our patients developed that upon follow-up. It is more likely that this represents a dysregulated immune response directed at the underlying *Bartonella* infection. Although the accuracy of provided patient histories in a pooled analysis of older literature can be significantly limited, the observed duration of IE was longer in cases with Bartonella IE than in cases caused by other microorganisms, suggestive of a subacute presentation (p<0.001), (**Table 1**).

The prevalence of ANCA positivity and low serum C3, and C4 were significantly higher in the cases with Bartonella IE-associated GN than those of IE-caused by other microorganisms. Although patients with other causes of IE maybe PR3-ANCA positive, PR3-ANCA positivity seems to be higher in Bartonella IE compared to other causes of IE (12), (**Table 1**).

Among cases with Bartonella IE-associated GN, 74 cases (83%) eventually fulfilled criteria for “definite IE” of the modified Duke criteria. However, some cases who had predisposing heart conditions underwent surgical treatment, such as valve replacement, even with the diagnosis of “possible IE” because of deteriorating cardiac function or uncontrolled fever. More details of the patient background in cases with Bartonella IE are shown in **Supplementary Table 1**.

#### 2.2.2 Summary of causative microorganisms

The confirmed causative microorganisms are shown in **Table 2** and **Supplementary Table 1**. Only four cases among those with Bartonella IE were blood culture positive. In contrast, all IE cases caused by other microorganisms were diagnosed based on the result of the blood culture. Most of the Bartonella IE cases were confirmed on blood serology, with anti-Bartonella henselae IgG, IgM, and/or anti-Bartonella quintana IgG and IgM. Some cases required polymerase chain reaction (PCR). However, the sensitivity of blood PCR was not high. Tissue-derived PCR (heart valve, lymph node, etc.) was performed in some cases, and the sensitivity was higher than that of blood PCR.

In the comparative group of 51 cases of culture-proven IE-related GN, the number of cases by causative microorganisms was as follows; methicillin-resistant Staphylococcus aureus (MRSA) (n=25), methicillin-sensitive Staphylococcus aureus (MSSA) (n=14), Staphylococcus (not specified)(n=3), Streptococcus species (n=4), and others; Enterococcus faecalis (n=3), methicillin-sensitive Staphylococcus epidermidis (n=1), Gram-positive cocci (n=1), Enterobacter (n=1), Peptostreptococcus (n=1), Anaerococcus prevotti (n=1), (few cases had multi-bacterial infection).

#### 2.2.3 Histological findings

The summary of histopathological findings and comparison of biopsy findings in Bartonella IE-associated GN with other IE-associated GN cases are shown in **Table 2**. The histopathological findings in the four in-house Bartonella cases are shown in more detail in **Table 5**.

#### 2.2.4 Light microscopy

Despite the limited data availability of detailed light microscopy findings in the published reports, we found that focal crescent formation is seen in significantly higher percentage of cases with Bartonella IE compared to other IE-associated GNs (83% versus 61%, p=0.006). However, we considered “any crescents” (we did not separate between cellular, fibrocellular, and fibrous crescents) because of the limited details provided in many of the published reports. Necrotizing lesions were also more prevalent in Bartonella-associated GN cases (P=0.02). Mesangial and endocapillary hypercellularity do not show significant differences from other IE-associated GNs. Occasional cases of Bartonella IE were reported to show a membranoproliferative (MPGN) pattern of injury as well (31).

#### 2.2.5 Immunofluorescence findings

**Figure 2:**
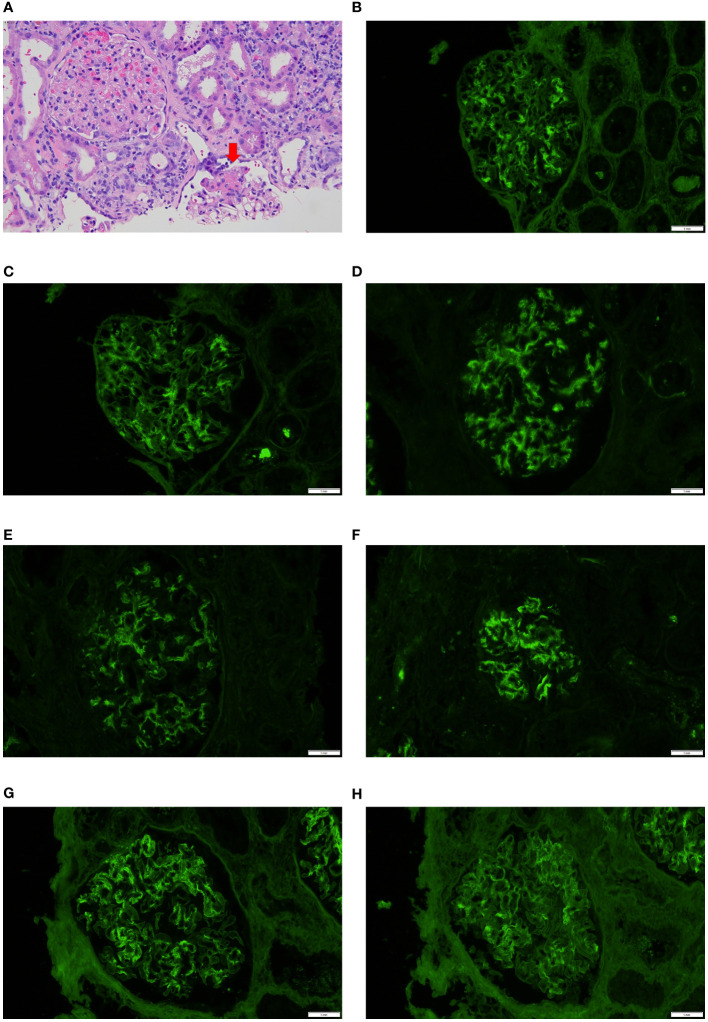
Biopsy features in Bartonella IE-associated glomerulonephritis **(A)**. Case 4 in-house case. Glomerulus on the bottom shows segmental necrotizing lesion (arrow) but the glomerulus on the left appears unremarkable, hematoxylin & eosin 40x; **(B–H)**. In-house Case 2 Direct immunofluorescence work-up showed “full-house pattern” with glomerular staining for IgG, IgA, IgM, C1q, C3, kappa and lambda (20x).

Few differences and some similarities in immunofluorescence findings between cases with Bartonella IE and cases with other microorganism-associated IE were seen. C1q and IgM positivity was higher in cases with Bartonella IE, on the other hand, IgA positivity was higher in cases of IE caused by other microorganisms, particularly Staphylococcus aureus IE. Two of the in-house cases of Bartonella IE and several from the pooled analysis (19, 24, 26, 31, 37, 47, 50, 81, 82) showed “full-house” IF pattern. However so did several cases from the other microorganisms-associated IE group, so this appears to be quite frequent in IE-associated GN, in general. Five cases of Bartonella IE from the pooled analysis and two of other microorganism-associated IE showed a “pauci-immune” pattern. One of the in-house cases of Bartonella IE-associated GN with a segmental necrotizing lesion is depicted in **Figure 2A**. Another case with “full-house” immunofluorescence pattern is shown in **Figures 2B–H**.

#### 2.2.6 Electron microscopy findings

There were no significant differences in electron microscopic findings between Bartonella IE and other microorganisms (**Table 3**). “Subepithelial hump” which is a term used to denote a large subepithelial electron dense deposit protruding outward, characteristic of PSAGN, is not commonly seen in other IRGNs and was uncommon in Bartonella IE-associated GN cases as well.

#### 2.2.7 Treatment

The data for cases with Bartonella IE only are shown here. We did not conduct a follow-up for IE caused by other microorganisms since it is beyond the scope of this study. Surgical treatment was conducted in 47/89 Bartonella IE-associated GN cases (53%). Rifampicin, doxycycline, and ceftriaxone were the most commonly used antibiotics. Gentamicin was also used to treat Bartonella IE, however, it was replaced by other antibiotics due to the concern for deteriorating renal function in some cases. The summary of treatment in cases with Bartonella IE is shown in **Supplementary Table 2**. Case 4 from our in-house cases of Bartonella IE, was initially managed with antibiotics alone. After a total of 6 months, doxycycline therapy was discontinued. The patient however developed disease relapse, requiring subsequent aortic and mitral valve replacement. PCR on the excised valvular tissue (but not on serum) was positive for *Bartonella henselae*.

Of note, at least 45 cases were shown to be treated with glucocorticoid (with/without) additional immunosuppressants, suggesting that the diagnosis of Bartonella IE-associated GN can be difficult and could be misdiagnosed as ANCA-associated GN or lupus nephritis. However, most cases halted or tapered glucocorticoid or immunosuppressants immediately after receiving positive serology results for Bartonella infection.

#### 2.2.8 Outcomes of Bartonella IE-associated GN cases

Regarding the outcomes of cases with Bartonella IE-associated GN, most cases showed improvement in general symptoms after surgical and/or antibiotic treatment. However, not all cases achieved recovery of normal renal function. Out of the 89 cases of Bartonella IE-associated GN, five cases had no follow-up, 47 cases underwent valve replacement surgery and 38 did not. The three cases that experienced retinal artery occlusion did not recover eyesight (22, 30, 35). Eight patients died within six months. Out of the four in-house cases, two died within a month of presentation.

### 2.3 Discussion

#### 2.3.1 Bartonella species in humans with IE

*Bartonella* spp. consist of small Gram-negative facultative intracellular bacilli with tropism for the endothelial cells, particularly the cardiac valvular endothelium of both the native as well as prosthetic valves, but can also involve adjacent structures of the valves, mural thrombus, and cardio-vascular devices. They can persist in the endothelial and subendocardial tissue (25). Up to 23 *Bartonella* spp. have been described, 11 of which are likely human pathogens. They were first recognized as endocarditis agents in 1993, the two most common agents being *B. henselae* and *B. quintana*. In fact, all cases in this pooled analysis, except one case were attributed to these two *Bartonella* spp. *Bartonella henselae* is known to be associated with cat-scratch disease, the most important risk factor being exposure to infected cats, especially young cats (25, 87), frequently affecting young individuals and commonly manifesting as a self-limited disease with rash, low grade fever, and enlarged tender regional lymph nodes. Other manifestations include disseminated disease manifested as bacteremia, endocarditis, ocular disease, encephalitis, bacillary angiomatosis, bacillary peliosis hepatis (the latter seen in the immunocompromised patients) (88–90). GN is an immune-mediated complication of endocarditis and although it is seen more frequently in older adults, it can affect all ages. Actually, 3 of 89 Bartonella IE cases in this pooled analysis were in their first decade, and two were in their second decade. *Bartonella quintana*, the agent of trench fever in the era of World war I, can also cause endocarditis and chronic bacteremia, reported commonly in the homeless and alcoholic patients with louse infestation (25, 87, 91). We found that, of 69 cases with *B. henselae* infection, 34 cases had a history of contact with cats (among our cases and as reported in the publications). Of 24 cases with *B. quintana* IE, only 5 patients reported heavy alcohol use, and 2 were homeless. Concomitant infection with both types of *Bartonella* spp. was reported in 16 cases, however this could have been a result of cross-reactivity on serological testing, a known phenomenon.

#### 2.3.2 Microbiologic diagnostic methods

*Bartonella* spp. are fastidious bacteria and difficult to grow in conventional culture. BCNE has been reported in 2.5 to 40% of IE cases and prevalence can vary depending on the geographic regions (92–95). Fournier PE et al. from France have reported a large series of BCNE (n=283 over a 5-year period) since they serve as a reference center for BCNE testing in the region (95). *Coxiella burnetti*, *Bartonella* spp. and *Topheryma whipplei* accounted for 15.9% of all BCNE cases, partly explained by the endemicity of Q-fever in that area. The other identified BCNE agents included enterococci, streptococci, staphylococci, the latter largely attributed to early administration of antibiotics, prior to blood culture collection. Other uncommon agents included *Legionella pneumophila*, *Mycobacterium bovis*, *Enterococcus fecalis* and *Enterococcus fecium and HACEK* spp. *(Hemophilus, Acinetobacter, Cardiobacterium, Eikenella and Kingella).* Serology using immunofluorescence assay is the easiest and most frequently used tool for laboratory diagnosis of Bartonella IE and considered the reference method despite cross-reactivity among Bartonella spp. and also with *Coxiella burnetti* and *Chlamydia* spp. Based on the review by Edouard et al. (9), Bartonella IgG titer of ≥1:800 has a positive predictive value of 95% in patients with IE (equivalent to >1:1024 with the assay commonly used in the United States). However these suggested cut-off values cannot be considered as rigid diagnostic criteria. A titer ≥1:800 was obtained in less than 60% of their cohort of patients with IE (9). A lower does not exclude the diagnosis of IE in patients with valvular disease and can be confirmed on Western blot analysis, which can offer a higher sensitivity (9). The IgG titers is considered more reliable than IgM titers. PCR assay on blood has a lower sensitivity than PCR on valvular tissue (9, 62, 63, 95). Based on the report by Fournier et al. (95), broad spectrum and specific PCR on blood samples detected pathogens in 3/177 (1.7%) and 24/177 (13.5%) patients of BCNE respectively. When applied to valvular specimens, broad range and specific PCR were positive in 52/119 (43.7%) and 45/119 (37.8%) patients respectively. Both broad range and specific PCR assays are available. Specific PCR assays target the most common pathogens in a given area and can potentially increase the diagnostic yield of BCNE, but are available only in selected laboratories. Warthin-Starry stain on tissue can highlight the bacteria, but is not specific (9, 95).

*Bartonella* spp. is reported as the second leading cause of BCNE after *Coxiella burnetti* (95), but that can vary by geographic region. Like Bartonella, *Coxiella burnetti* also shows tropism for the endothelium and endocardial tissue and can similarly predispose to autoimmune phenomena such as vasculitis, but in contrast to Bartonella, the latter shows predominantly involvement of medium to large size vessels (mimicking polyarteritis nodosa, giant cell arteritis and Takayasu’s arteritis respectively). Small vessel vasculitis manifesting as cryoglobulinemic GN was seen in 75% of the Coxiella infection cases and 8% of the Bartonella infected patients (16). ANCA-associated vasculitis (AAV) was seen in 13% of Coxiella and 83% of Bartonella patients in the same series. ANCA positivity was also more common with Bartonella versus Coxiella. Generally, *Hemophilus* spp., *Aggregatibacter actinomycetemcomitans*, *Cardiobacterium hominis*, *Eikenella corrodens*, and *Kingella kingae* (the *HACEK* organisms) are other fastidious causative organisms for BCNE (96). In several of the Bartonella cases reported in the described series, antibiotics for targeting *HACEK* group of microorganisms were also given as part of initial management (31, 44, 63, 82). However none of our cases with Bartonella IE nor cases from the pooled analysis showed concomitant *Coxiella* or *HACEK* infections.

#### 2.3.3 Guidelines for diagnosis of Bartonella IE-associated GN and distinguishing from other microorganisms causing IE

Diagnosis may be easy in the presence of classical features such as fever, cardiac murmer, bacteremia (positive cultures), characteristic valvular vegetations on echocardiography with peripheral vascular stigmata. However in routine practice, variations may be seen.

The higher frequency of positive ANCA serology positivity (with proteinase-3 specificity/cANCA), higher frequency of purpuric skin rash, active glomerular crescents and necrotizing lesions on the kidney biopsy, and lack of positive blood cultures due to the fastidious nature of these Gram-negative bacilli increases the chances of misdiagnosis as ANCA-associated GN. Frequent glomerular IgM and C1q staining and sometimes “full-house” immunofluorescence pattern also brings lupus nephritis in the differential diagnosis, particularly in younger patients. This heightens the urgency for correct diagnosis and treatment and to avoid inadvertent immunosuppressive drug regimens. This is often challenging since IE is like a “syndrome” with a broad spectrum of findings and multi-organ involvement, the reasons being - high propensity for heart failure in IE, and associated embolic phenomena. It requires a multi-disciplinary approach and is therefore relevant to a broad spectrum of medical providers besides nephrologists. The presence of protracted non-specific generalized symptoms, cardiovascular symptoms, pancytopenias, splenomegaly, along with a nephritic pattern of renal failure should raise strong suspicion for possible IE, leading to specific evaluation using the updated modified Duke criteria, particularly transesophageal echocardiography (TEE), review of pre-existing valvular disease, valvular surgery or other prosthetic devices in the heart (13, 14). Although a small percentage (16%) of *Staphylococcus aureus* IE also reported a history of prior valvular abnormalities, they tended to report other risk factors mainly IDU, which causes direct blood stream infection capable of affecting previously intact heart valves. Taking this into account, the minor modified Duke criterion of the pre-existing cardiac issues can be very helpful in the early diagnosis of Bartonella-associated IE (13, 14). The aortic valve is most frequently involved (65%) followed by the mitral valve (31%), in contrast to *Staphylococcus aureus* IE in patients with IDU, which more frequently affects the tricuspid valve due to direct blood stream infections (2, 5).

A serologic work-up is critical to include in the diagnostic test battery and for that, it very important to keep in mind infection in the differential diagnosis of these critically-ill patients. Positive serology for *Bartonella* spp. is in fact has been added as one of the major Duke criteria for diagnosis of IE, per the most updated guidelines from 2023 (14). History of animal exposure can be a very important clue to diagnosis and is often missed during initial patient encounter.

Bartonella IE patients were on average older by a decade compared to IE with other microorganisms, with an even greater male predominance longer estimated time to biopsy, presence of additional immune serologies including rheumatoid factor and more frequent low serum C3 levels. C3-dominant glomerular staining is common on biopsy similar to SAGN, but IgA staining is much less frequent in contrast to SAGN. Purpuric skin rash was found more frequently in Bartonella IE-associated GN based on the estimates provided in the published reports. The incidence may be even higher if the rash was transient and/or not a prominent presenting symptom.

#### 2.3.4 Distinguishing Bartonella IE-associated GN from true ANCA-associated vasculitis

Absence of upper respiratory tract involvement, extracardiac manifestations limited to the skin (mostly purpura) and kidney, along with splenomegaly, hypocomplementemia (low serum C3 and C4), dual ANCA positivity, low ANCA titers, C1q, IgM and other immune reactants in the glomerular immune complex deposits, presence of other autoantibodies like anti-nuclear antibody, rheumatoid factor are all important distinguishing clues and are encountered much more commonly in IE-associated GN than in AAV. AAV often has pulmonary and central nervous system findings, while splenomegaly is rare (11, 12). Pulmonary nodules are described in up to 73% of cases with true ANCA vasculitis/GN (16). Other bacteria (such as Staphylococcus aureus, Streptococcus pneumoniae) with a higher tendency to develop lung infection/abscess can however be accompanied by pulmonary nodules/consolidations (unlike Bartonella species). We have also reported a case of pulmonary-renal syndrome with hemoptysis in MRSA-associated GN (97).

#### 2.3.5 Guidelines for treatment of Bartonella IE-associated GN

**Figure 3:**
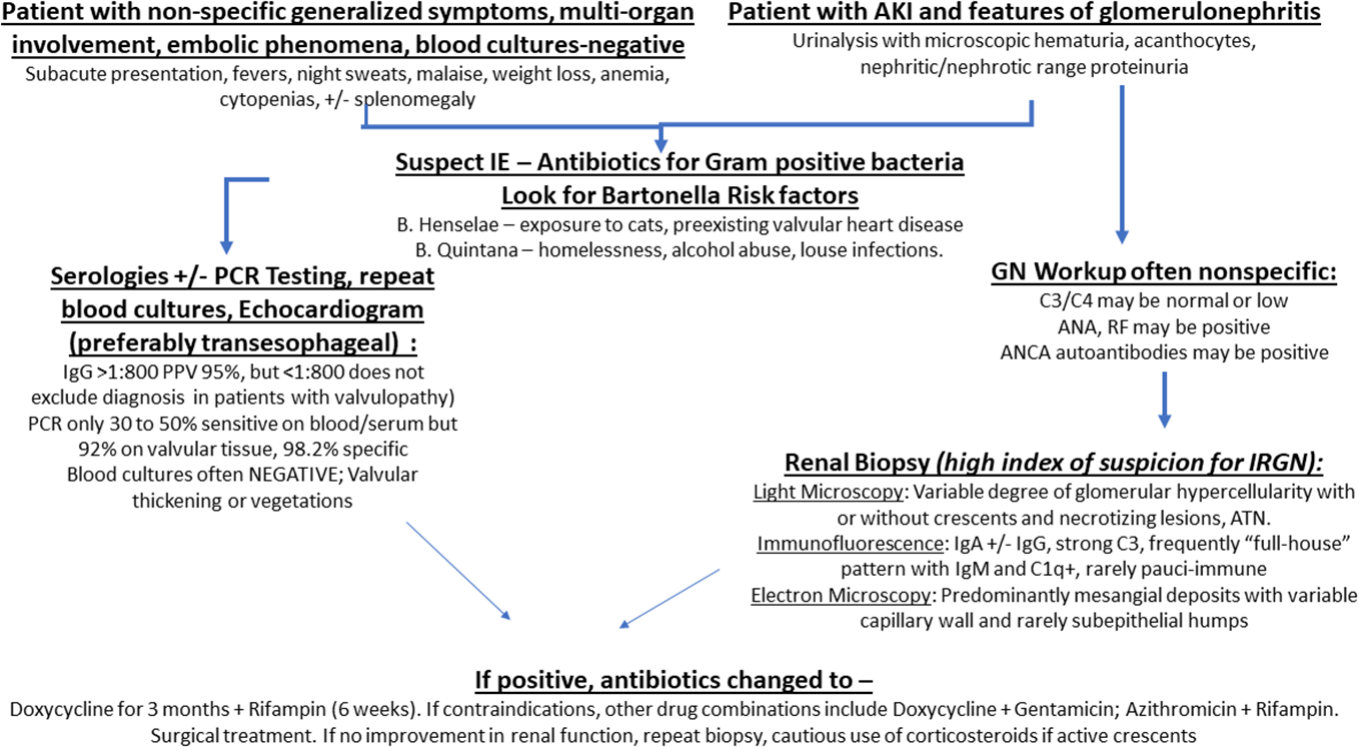
Flowchart depicting management protocol for Bartonella IE-associated GN.

Readers may refer to more detailed texts for elaborate and current treatment protocols (98, 99). A flowchart is shown in **Figure 3**. In brief, the preferred regimen for Bartonella IE includes a combination of Doxycycline (3 months) along with Rifampin (6 weeks). If any of these are contraindicated, alternative combinations consist of Azithromycin with Rifampin; or Doxycycline with Gentamycin. Ceftriaxone is usually added to above antibiotics in cases of “culture-negative” IE where Bartonella is “suspected”, but not “proven”, but its primary role is to cover for other pathogens that can cause “culture-negative IE”. Once Bartonella is confirmed, Ceftriaxone can be stopped. These antibiotics can be administered irrespective of the renal function. Prolonged courses up to 3 months or more are recommended (98). Ongoing inflammation, necrosis and bacteria in excised damaged cardiac valves have been demonstrated even after few weeks of antibiotic treatment (19). Valvular excision (and replacement) is frequently necessary to remove the nidus of infection in addition to medical management to restore normal valvular function. The timing of the surgery varied depending on time to diagnosis (which in some cases is prolonged because of persistently negative blood cultures and low index of suspicion for underlying infection), the patient’s clinical status and also because inadvertently given immunosuppression therapy (mainly corticosteroids for presumed ANCA-associated GN), needs to be tapered. Nonetheless, almost all patients who underwent surgical treatment, such as valve replacement therapy showed improvement in renal function after surgery. Some cases were able to control Bartonella infection with antibiotics alone, under careful monitoring with echocardiography, but bacterial cure may be difficult to achieve as demonstrated in Case 4 (**Table 4**) who had relapse following discontinuation of antibiotic therapy (administered for 5 months) requiring surgical replacement of the pre-existing prosthetic aortic and mitral valve.

Treatment for the GN is important and mainly involves supportive care including management of hypertension and fluid overload with diuretic therapy and salt restriction. Calcium channel blockers are also preferred as antihypertensive drugs but, in patients with stable renal function, ACE inhibitors and angiotensin receptor blockers might be an alternative option. Dialysis might also be necessary to correct electrolyte and metabolic disturbances. Immunosuppression should be avoided during active infection similar to SAGN as it might impede clearance of the active infection and increase the risk of death (2, 6). But there is always the issue of what to do after the infection has been eradicated but renal dysfunction persists. One possibility is to biopsy the patient after they have completed an appropriate course of antibiotics to determine if nephritis is still active. Renal dysfunction might persist due to permanent kidney damage (widespread glomerular sclerosis/scarring due to crescentic GN; interstitial fibrosis) sustained while the infection was still active. In cases with persistent inflammation and/or cellular crescents/necrotizing glomerular lesions despite bacterial clearance, a course of corticosteroids could be considered to limit further damage to the kidneys but should be used with caution and close monitoring for infection relapse.

Although we did not include such patients in this study, organ transplant recipients are also vulnerable to Bartonella (100). Several renal allograft recipients have been reported to have Bartonella IE (91). Of note, some kidney transplant recipients with Bartonella IE showed thrombotic microangiopathy, which is not reported in native kidney biopsies (101).

Our review has critical biases. Given the nature of a literature-based review, it is important to acknowledge that our assessment may not fully mitigate inherent biases as the most complex are usually reported as opposed to the less striking ones. So prevalence of ANCA positivity, frequency of crescents may be overemphasized. Data availability varied among the cases. For instance, nephrologists and pathologists tended to provide extensive information on pathological findings, whereas doctors specializing in infectious diseases focused on microorganisms and diagnostic methods. In an effort to offer a comprehensive view of pathological findings from the perspective of pathologists, we have included detailed information from our in-house cases.

### 2.4 Conclusion

Bartonella IE-associated GN is on the rise since Bartonella has emerged as one of the leading causes of BCNE, among others such as *Coxiella burnetti* and *HACEK* group of bacteria. The number of cases of Bartonella IE-associated GN in single centers has been small (relative to SAGN), and diagnosis is often least suspected. To our knowledge, ours is the first report to highlight the clinicopathological differences between Bartonella IE-associated GN versus IE caused by other bacteria. Although overall histologic patterns do overlap with other forms of IRGN, in particular SAGN, Bartonella IE-associated GN does show a higher frequency of crescents/necrotizing lesions in the biopsy which along with frequently positive ANCA serology can easily mimic ANCA- associated GN. Also, a ‘full-house” immunofluorescence staining pattern (particularly IgM and C1q) may occur and, along with multiple positive autoimmune serologies, can mimic lupus nephritis. Culture negativity, nonspecific generalized symptoms, multi-organ involvement or localized organ damage due to embolic phenomena can together make diagnosis quite challenging. Targeted history of animal exposure, particularly cats, and serology work-up for *Bartonella* spp. particularly in patients with congenital heart disease, prior valvular disease and/or valvular surgery are two important things that can help expedite clinical diagnosis. Prolonged antibiotic courses are needed, but valvular excision and replacement surgery may become necessary for bacterial cure.

## Author contributions

MK: Data curation, Formal analysis, Investigation, Methodology, Writing – original draft, Writing – review & editing. AD: Data curation, Writing – original draft. JH: Data curation, Writing – original draft. SP: Writing – review & editing. TN: Writing – review & editing. EC: Validation, Writing – review & editing. JB: Validation, Writing – original draft. AS: Conceptualization, Investigation, Project administration, Supervision, Validation, Writing – original draft, Writing – review & editing, Data curation, Methodology, Resources.
