# Supplementary material for: Clinicopathological differences between Bartonella and other bacterial endocarditis-related glomerulonephritis – our experience and a pooled analysis
Source: Front Nephrol. 2024 Jan 15;3:1322741. doi: 10.3389/fneph.2023.1322741 (PMC10823370; doi:10.3389/fneph.2023.1322741)
Supplement: Supplementary file 1 [file Table_1.docx]

Supplementary Material

**Supplementary Table 1. Demographic features of Bartonella endocarditis cases**

| **Ref No** | **Year** | **Age** | **Sex** | **Animal contact history** | **Prior history**  **of valvular**  **disease** | **Other risk factors** | **s-Cr** | **Low**  **C3** | **Low**  **C4** | **ANCA** | **ANA** | **Confirmation of Bartonella infection** | **Micro-organisms** | **Duke criteria** | **Location of IE** | **Other IE complications** |
| --- | --- | --- | --- | --- | --- | --- | --- | --- | --- | --- | --- | --- | --- | --- | --- | --- |
| 15 | 2022 | 67 | M | N/A | N/A | N/A | N/A | Yes | Yes | PR3+ | Neg | Se(B) | B. quintana | >Possible* | N/A |  |
| 15 | 2022 | 78 | M | N/A | N/A | N/A | N/A | Yes | No | PR3+ | Neg | Se(B), PCR(T)  Blood culture+ | B. henselae | Definite | A |  |
| 15 | 2022 | 48 | F | N/A | N/A | N/A | N/A | No | No | PR3+ | Neg | Se(B) | B. henselae | Definite | N/A | skin lesion |
| 15 | 2022 | 54 | M | N/A | N/A | N/A | N/A | No | No | PR3+ | Neg | Se(B), PCR(T) | B. henselae | >Possible* | A |  |
| 15 | 2022 | 72 | F | N/A | N/A | N/A | N/A | Yes | Yes | Neg | Neg | Se(B) | B. spp | >Possible* | N/A | skin lesion |
| 16 | 2022 | 17 | F | cat | pulmonary atresia, VSD, AVR | None | 1.45 | No | Yes | PR3+ | Neg | Tissue culture+ | B. spp | Definite | A | septic PE susp,  skin lesion |
| 17 | 2022 | 52 | M | cat | rheumatic fever+,  MS, AR | None | 3.91 | Yes | N/A | PR3+ | Neg | Se(B), PCR(B) | B. henselae | Definite | A |  |
| 18 | 2022 | 13 | F | no history | bicuspid AV | None | 2.5 | Yes | Yes | Neg | Neg | Se(B), PCR(T) | B. henselae | Definite | A | skin lesion |
| 19 | 2022 | 43 | M | cat | ascending aortic aneurysm, AVR | None | N/A | Yes | No | PR3+ | Neg | Se(B), PCR(B) | B. quintana | Possible | A | skin lesion |
| 20 | 2022 | 39 | M | cat | MVP | None | 4.5 | N/A | N/A | N/A | N/A | Se(B), WS stain | B .spp | Definite | M |  |
| 20 | 2022 | 58 | M | cat | AVR | None | 3 | N/A | N/A | atypical+ | N/A | Se(B), PCR(B), WS stain | B. spp | Definite | A |  |
| 20 | 2022 | 69 | M | cat | no history | None | 3.5 | N/A | N/A | N/A | N/A | Se(B) | B. spp | Definite | A, M, T | arterial emboli |
| 21 | 2022 | 66 | M | cat | no history | None | 2.6 | N/A | N/A | Neg | N/A | Se(B),  blood culture+ | B. henselae | Definite | M | retinal artery occlusion |
| 22 | 2021 | 48 | M | cat | no history | None | N/A | Yes | Yes | N/A | N/A | Se(B), PCR(T),  WS stain | B. henselae | Definite | M |  |
| 23 | 2021 | 75 | M | cat | AV anomaly | drug abuse  homeless | 2.4 | Yes | Yes | Neg | Neg | Se(B) | B. henselae  B. quintana | Definite | A | skin lesion |
| 24 | 2021 | 65 | M | no history | AR, MR | alcoholic | 2.69 | No | No | PR3+MPO+ | Neg | Se(B), WS stain | B. spp | Definite | A, M | cerebral aneurysm,  skin lesion |
| 25 | 2021 | 70 | F | cat | None | None | 7.81 | Yes | No | PR3+ | Pos | PCR(B) | B. henselae | Possible | A |  |
| 26 | 2021 | 20 | M | dog | Noonan syn, PS, MS | None | 2.9 | N/A | N/A | N/A | N/A | Se(B), PCR(B,T),  immunobiological stain | B. henselae  B. quintana | Definite | M | cerebral aneurysm |
| 27 | 2021 | N/A | N/A | cat | AS | None | N/A | Yes | Yes | N/A | Pos | Se(B), PCR(B,T) | B. henselae | Definite | A | embolic stroke |
| 27 | 2021 | N/A | N/A | cat | None | None | N/A | Yes | Yes | cANCA+ | N/A | Se(B) | B. henselae | Definite | A |  |
| 28 | 2020 | 36 | M | animals | bicuspid AV | alcoholic | 7.3 | Yes | Yes | PR3+ | Pos | Se(B), PCR(T) | B. quintana | Definite | A,T | skin lesion |
| 29 | 2020 | 66 | M | cat | no history | None | 8.3 | Yes | Yes | Neg | Neg | Se(B) | B. henselae  B. quintana | Definite | M, T | retinal artery occlusion |
| 30 | 2020 | 19 | M | cat | PVR | None | 3.44 | Yes | Yes | Neg | Pos | Se(B), PCR(B,T) | B. henselae | Definite | P |  |
| 31 | 2020 | 63 | M | cat | no history | alcoholic | 3.9 | No | No | atypical+ | Neg | Se(B), PCR(B,T),  pathologic examination | B. henselae | Possible | A | skin lesion |
| 32 | 2020 | 8 | M | cat | pulmonary atresia | None | N/A | Yes | Yes | N/A | Pos | Se(B) | B. henselae | Definite | P |  |
| 33 | 2020 | 38 | M | cat | AVR | alcoholic | 5.61 | Yes | No | PR3+ | Neg | Se(B), PCR(T) | B. henselae | Definite | A | bilateral papilledema |
| 34 | 2019 | 66 | F | cat | AVR | None | 2.74 | N/A | N/A | cANCA+ | Pos | Se(B) | B. henselae | Possible | N/A | retinal artery occlusion, skin lesion |
| 35 | 2019 | 32 | M | cat | truncus arteriosus, AVR | None | 5.4 | Yes | Yes | MPO+ | Neg | PCR(B) | B. spp | Possible | N/A | skin lesion |
| 36 | 2019 | 54 | M | cat, dog | AVR | None | 3.2 | N/A | N/A | PR3+ | Neg | Se(B) | B. henselae | Definite | A |  |
| 37 | 2018 | 47 | M | no history | bicuspid AV | None | 2.36 | No | No | PR3+ | Neg | tissue culture+,  specialized stain | B. henselae | Definite | A |  |
| 38 | 2018 | 48 | M | cat, dog | AVR, PMI | lice infection | N/A | Yes | Yes | Neg | N/A | Se(B), PCR(B,T) | B. henselae | Definite | A | skin lesion |
| 39 | 2018 | 29 | M | cat | complete transition of the great arteries and VSD | None | 3.3 | Yes | Yes | Pos  (not specified) | N/A | Se(B) | B. henselae | Definite | P |  |
| 40 | 2017 | 55 | M | cat | no history | alcoholic | 2.65 | Yes | Yes | Neg | Neg | Se(B) | B. henselae | Definite | A | intracranial aneurysm. skin lesion |
| 41 | 2017 | 61 | M | animals | AVR | None | 2.22 | N/A | N/A | PR3+ | Neg | Se(B) | B. henselae | Definite | A | mycotic aneurysm,  skin lesion |
| 42 | 2017 | 64 | F | N/A | no history | None | 0.82 | No | No | pANCA+ | N/A | PCR(T)  blood culture+ | B. bacilliformis | Definite | T | skin lesion |
| 43 | 2017 | 67 | M | animals | no history | None | 4.35 | Yes | No | cANCA+ | Pos | Se(B) | B. henselae | Definite | A, M |  |
| 44 | 2017 | 62 | M | cat | multiple surgery, AVR | None | 2.76 | N/A | N/A | PR3+ | N/A | Se(B), PCR(B) | B. henselae  B. quintana | Definite | A | subconjunctival hemorrhages |
| 45 | 2016 | 55 | M | cat | no history | alcoholic  homeless | 5.51 | Yes | No | PR3+ | Neg | Se(B) | B. henselae  B. quintana | Definite | A |  |
| 46 | 2016 | 6 | M | cat | ASD, VSD, multiple congenital anomaly | None | 1.14 | N/A | N/A | PR3+ | N/A | Se(B), PCR(T) | B. henselae | Definite | P |  |
| 46 | 2016 | 14 | M | cat | multiple congenital anomaly | None | 2.01 | N/A | N/A | cANCA+ | N/A | Se(B), PCR(T) | B. henselae | Definite | P | septic PE, skin lesion |
| 47 | 2016 | 42 | F | animals | no history | drug abuse | 2.29 | Yes | No | PR3+, MPO+ | Pos | Se(B), PCR(T),  Steiner stain | B. henselae | Definite | T | skin lesion |
| 48 | 2016 | 21 | M | cat | multiple congenital anomaly | None | 3.51 | Yes | Yes | Neg | Neg | Se(B), PCR(T) | B. henselae | Definite | P | presyncope episodes |
| 49 | 2016 | 45 | M | cat | bicuspid AV | None | 2.99 | N/A | N/A | PR3+ | Neg | Se(B), PCR(T),  WS stain | B. henselae | Definite | A | skin lesion |
| 50 | 2015 | 67 | M | cat, dog | bicuspid AV | None | 3.5 | Yes | No | MPO+, PR3+ | Neg | Se(B) | B. henselae | Definite | A, M | skin lesion |
| 51 | 2015 | 54 | M | no history | no history | alcoholic | 4.79 | Yes | Yes | N/A | Pos | Se(B), WB | B. quintana | Definite | A, M | skin lesion |
| 52 | 2015 | 9 | M | cat | DiGeorge syn, TOF, multiple surgeries | None | 5.3 | Yes | No | N/A | N/A | Se(B) | B. henselae | Definite | T |  |
| 53 | 2014 | 18 | F | cat | TOF, multiple surgeries | None | 1.48 | Yes | Yes | PR3+ | Pos | Se(B), PCR(B) | B. spp | Possible | N/A |  |
| 54 | 2014 | 36 | M | cat | bicuspid AV | None | 2.1 | N/A | N/A | PR3+ | N/A | Se(B) | B. henselae | Definite | A | skin lesion |
| 55 | 2013 | 78 | F | no history | no history | None | 2.21 | Yes | No | PR3+ | Pos | Se(B) | B. henselae | Definite | A | mycotic aneurysm,  skin lesion |
| 56 | 2012 | 74 | M | no history | no history | None | 2.43 | Yes | Yes | PR3+ | Neg | Se(B) | B. henselae | Definite | A, T | septic PE susp |
| 57 | 2011 | 74 | M | cat | AVR | None | 5.5 | Yes | Yes | PR3+ | Neg | Se(B) | B. henselae | Definite | A | vasculitic change in brain, skin lesion |
| 58 | 2011 | 59 | M | cat | AVR | None | 5.5 | N/A | N/A | N/A | N/A | PCR(B, T),  Tissue culture+,  WS stain | B. henselae | Definite | A | skin lesion |
| 59 | 2009 | 64 | M | no history | no history | None | 1.33 | Yes | Yes | cANCA+ | Pos | Se(B) | B. quintana | Possible | A | skin lesion |
| 60 | 2009 | 72 | M | no history | no history | None | N/A | N/A | N/A | Neg | Neg | Se(B), PCR(T) | B. quintana | Definite | A, T |  |
| 61 | 2007 | 43 | M | cat | MVR, AVR | None | N/A | N/A | N/A | PR3+ | N/A | Se(B), PCR(T) | B. henselae | Definite | A,M |  |
| 62 | 2006 | 45 | M | no history | MVR, AVR | None | 3.8 | N/A | N/A | N/A | N/A | Se(B), PCR(T),  blood culture+ | B. quintana | Definite | A, M | splenic infarction, cerebral hemorrhage |
| 63 | 2005 | 36 | M | no history | AVR | drug abuse  alcoholic | 6.3 | N/A | N/A | N/A | N/A | Se(B), PCR(T),  Giemsa stain | B. henselae  B. quintana | Definite | M | nothing particular |
| 63 | 2005 | 76 | M | no history | MVR, AVR | alcoholic | 8 | N/A | N/A | N/A | N/A | Se(B) | B. henselae  B. quintana | Definite | M |  |
| 64 | 2005 | 58 | M | cat | bicuspid AV | None | 4.57 | N/A | N/A | PR3+ | N/A | Se(B), PCR(T) | B. henselae | Definite | M | AMI |
| 65 | 2005 | 79 | M | no history | AVR | None | 4.52 | N/A | N/A | N/A | Pos | Se(B), PCR(B),  WB | B. spp | Definite | A |  |
| 66 | 2004 | 69 | M | no history | no history | None | 3.39 | N/A | N/A | N/A | N/A | Se(B), PCR(B) | B. henselae B. quintana | Possible | M |  |
| 67 | 2004 | 53 | F | dog | rheumatic heart disease (MR,TR) | None | 3.5 | Yes | Yes | Neg | Neg | Se(B) | B. henselae | Definite | M |  |
| 67 | 2004 | 35 | M | cat | bicuspid AV | None | 4.5 | No | Yes | Neg | Neg | Se(B) | B. henselae | Definite | A |  |
| 67 | 2004 | 46 | M | N/A | no history | alcoholic  homeless | 5.2 | Yes | Yes | Neg | Neg | Se(B) | B. henselae | Definite | A, M |  |
| 68 | 2001 | 53 | M | pets | bicuspid AV | None | 2.05 | N/A | N/A | Neg | Neg | Se(B) | B. spp | Definite | A |  |
|  |  |  |  |  |  |  |  |  |  |  |  |  |  |  |  |  |
| 69 | 2022 | 74 | M | no history | no history | None | 2.1 | Yes | No | MPO+ | Pos | Se(B), PCR(B),  WS stain | B. henselae | Definite | A |  |
| 70 | 2022 | 66 | M | cat | no history | None | 2.6 | N/A | N/A | N/A | N/A | Se(B) | B. henselae  B. quintana | Definite | T | left eye blindness |
| 71 | 2021 | 33 | M | no history | no history | HIV+ | 3.3 | No | Yes | PR3+ | N/A | Se(B), PCR(T) | B. henselae  B. quintana | Definite | A | cerebral artery aneurysm |
| 72 | 2021 | 44 | M | no history | bicuspid AV | None | >4 | N/A | N/A | PR3+ | N/A | Se(B) | B. henselae  B. quintana | Definite | A |  |
| 73 | 2020 | 62 | M | no history | rheumatic heart disease | None | 2.61 | N/A | N/A | PR3+ | N/A | PCR(T) | B. henselae | Definite | A |  |
| 74 | 2020 | 71 | M | no history | AVR | None | N/A | Yes | Yes | PR3+ | N/A | Se(B), PCR(T) | B. henselae  B. quintana | Definite | A, M | skin lesion |
| 75 | 2019 | 56 | M | no history | left ventricular assist device | None | 3.45 | No | No | PR3+ | Pos | Se(B) | B. henselae | Definite | others |  |
| 75 | 2019 | 42 | M | no history | AVR | None | 4 | Yes | Yes | MPO, PR3+ | Pos | Se(B), PCR(T) | B. henselae  B. quintana | Definite | A | skin lesion |
| 75 | 2019 | 64 | M | no history | MVR | None | 6 | Yes | No | Neg | Neg | Se(B) | B. henselae  B. quintana | Definite | M |  |
| 76 | 2019 | 31 | F | no history | pulmonary atresia (Down syn.) | None | N/A | Yes | Yes | N/A | N/A | Se(B) | B. henselae | Possible | N/A | skin lesion |
| 77 | 2018 | 63 | M | no history | AVR | None | 3.8 | Yes | No | PR3+ | Neg | Se(B) | B. henselae | Definite | A |  |
| 78 | 2018 | 52 | M | no history | AVR | None | 7 | N/A | N/A | Neg | Neg | Se(B), PCR(T) | B. henselae | Definite | A | AMI |
| 79 | 2018 | 73 | M | no history | no history | None | 5.9 | Yes | No | PR3+ | N/A | Se(B) | B. henselae | Definite | T | Septic PE |
| 80 | 2018 | 79 | M | cat | AVR, MVR | None | 2.14 | Yes | Yes | PR3+ | N/A | Se(B) | B. henselae  B. quintana | Definite | A,M |  |
| 81 | 2018 | 54 | M | no history | rheumatic fever+,  AR | None | 2.5 | N/A | N/A | N/A | N/A | Se(B) | B. henselae | Possible | N/A |  |
| 82 | 2017 | 56 | M | no history | AVR | None | 3.2 | N/A | N/A | N/A | N/A | Se(B), PCR(B) | B. quintana | Definite | A |  |
| 83 | 2016 | 55 | M | no history | no history | alcoholic | 5.5 | Yes | No | PR3+ | Neg | Se(B) | B. henselae  B. quintana | Definite | A,M |  |
| 84 | 2014 | 20 | M | animals | AVR, PVR | None | 3.1 | Yes | Yes | N/A | N/A | tissue pathology | B. henselae | Definite | P |  |
| 85 | 2007 | 37 | M | no history | MVR | drug abuse | 6.2 | No | No | pANCA+ | N/A | Se(B), PCR(T),  WS stain | B. henselae  B. quintana | Definite | M |  |
| 86 | 2001 | 65 | M | no history | rheumatic heart disease | None | 2.0 | No | Yes | N/A | N/A | PCR(T),southern blotting | B. henselae | Definite | N/A | ischemic stroke |
| Our | N/A | 74 | M | no history | AVR | None | 2.5 | Yes | Yes | PR3+ | Neg | Se(B) | B. henselae | Definite | M | splenic infarction, skin lesions |
| Our | N/A | 68 | M | cat | AVR, MVR | None | 3.46 | Yes | Yes | Neg | Pos | Se(B), PCR(B) | B. henselae | Definite | A | skin lesions |
| Our | N/A | 64 | M | no history | aortic aneurysm repaired | None | 5.8 | No | No | MPO+ PR3+ | Neg | Se(B), PCR(B) | B. henselae | Possible | N/A | skin lesions |
| Our | N/A | 79 | M | cat | AVR | None | 4.2 | Yes | Yes | PR3+ | Neg | Se(B) | B. henselae | Definite | A |  |

* The reference paper stated that endocarditis existed.

M, male; F, female; N/A, not available; VSD, ventricular septal defect; AVR, aortic valve replacement; MS, mitral stenosis; AR, aortic regurgitation; AV, aortic valve; MVP, mitral valve prolapse; MR, mitral regurgitation; PS, pulmonary stenosis; PVR, pulmonic valve replacement; PMI, pacemaker implantation; ASD, atrial septal defect; TOF, tetralogy of Fallot; TR, tricuspid regurgitation; MVR, mitral valve replacement; s-Cr, serum creatinine; ANCA, antineutrophil cytoplasmic antibodies; ANA, antinuclear antibody; Se(B), serology blood; PCR(B), polymerase chain reaction blood; PCR(T), polymerase chain reaction tissue; WS stain, Warthin-Starry stain; WB, western blotting;

**Supplementary Table 2. Clinical course of Bartonella endocarditis cases**

| **Ref No** | **Age** | **Sex** | **antibiotics**  **for Bartonella** | **surgical treatment** | **steroid, IS** | **Cr at bx** | **Renal outcomes**  **(Boils 2015)** |
| --- | --- | --- | --- | --- | --- | --- | --- |
| 15 | 67 | M | AMPC, DOXY, GM | Unknown |  | N/A | N/A |
| 15 | 78 | M | DOXY, RFP | Yes | GC, CYC, PLEX | N/A | N/A |
| 15 | 48 | F | DOXY, GM | Unknown |  | N/A | N/A |
| 15 | 54 | M | AMPC, GM, vibramycin | Yes | GC, CYC, PLEX | N/A | N/A |
| 15 | 72 | F | GM, TAZ/PIPC, spiramycin | Unknown |  | N/A | N/A |
| 16 | 17 | F | GM, RFP, DOXY | Yes | GC | 1.45 | CR |
| 17 | 52 | M | DOXY, GM | No | GC | 3.91 | PRD |
| 18 | 13 | F | DOXY, GM | Yes |  | 2.5 | CR |
| 19 | 43 | M | DOXY, RFP | No | GC | N/A | PRD |
| 20 | 39 | M | Antibiotics | Yes |  | 4.5 | N/A |
| 20 | 58 | M | Antibiotics | Yes | GC | 3 | N/A |
| 20 | 69 | M | Antibiotics | Yes |  | 3.5 | N/A |
| 21 | 66 | M | DOXY, RFP | No |  | 2.6 | N/A |
| 22 | 48 | M | DOXY, RFP | Yes |  | N/A | PRD |
| 23 | 75 | M | DOXY, RFP | No | GC, RTX | 2.4 | PRD |
| 24 | 65 | M | CTRX, DOXY, GM | No |  | 2.69 | CR |
| 25 | 70 | F | DOXY, RFP | Yes |  | 7.81 | ESRD |
| 26 | 20 | M | CTRX, DOXY, RFP | Yes |  | 2.9 | CR |
| 27 | N/A | N/A | CTRX, DOXY, GM, ST | Yes | GC, MMF | N/A | N/A |
| 27 | N/A | N/A | DOXY, GM, RFP | No | GC, MMF | N/A | N/A |
| 28 | 36 | M | DOXY, RFP | Yes |  | 7.3 | N/A |
| 29 | 66 | M | DOXY, RFP | No | GC | 8.3 | PRD |
| 30 | 19 | M | DOXY, RFP | Yes | GC, HQ | 3.44 | ESRD |
| 31 | 63 | M | DOXY, GM, RFP | Yes | GC, MMF | 3.9 | CR |
| 32 | 8 | M | CTRX, DOXY, GM | Yes | GC, MMF | N/A | N/A |
| 33 | 38 | M | DOXY, RFP | Yes | GC, CYC | 5.61 | N/A |
| 34 | 66 | F | DOXY, GM | No | GC | 2.74 | CR |
| 35 | 32 | M | DOXY, RFP | No |  | 5.4 | ESRD |
| 36 | 54 | M | CTRX, DOXY, GM | No | GC | 3.2 | N/A |
| 37 | 47 | M | DOXY, RFP | Yes |  | 2.36 | PRD |
| 38 | 48 | M | DOXY, RFP | Yes | GC, PLEX | N/A | PRD |
| 39 | 29 | M | CTRX, DOXY | Yes | GC | 3.3 | PRD |
| 40 | 55 | M | DOXY, GM | No | GC, CYC | 2.65 | PRD |
| 41 | 61 | M | DOXY | Yes |  | 2.22 | CR |
| 42 | 64 | F | CPFX, GM, RFP | No | GC, AZA | 0.82 | N/A |
| 43 | 67 | M | DOXY, RFP | No | GC | 4.35 | PRD |
| 44 | 62 | M | DOXY, GM | No |  | 2.76 | PRD |
| 45 | 55 | M | DOXY, GM, RFP | No | GC | 5.51 | CR |
| 46 | 6 | M | CTRX, DOXY, RFP | Yes |  | 1.14 | N/A |
| 46 | 14 | M | DOXY, RFP | Yes |  | 2.01 | N/A |
| 47 | 42 | F | CTRX, DOXY, GM | Yes | GC | 2.29 | N/A |
| 48 | 21 | M | DOXY, RFP | Yes |  | 3.51 | CR |
| 49 | 45 | M | DOXY, MEPM, aminoglycoside | Yes | GC, CYC, MPA | 2.99 | PRD |
| 50 | 67 | M | DOXY, RFP | Yes | GC, CYC | 3.5 | PRD |
| 51 | 54 | M | AMPC, DOXY, GM | No |  | 4.79 | PRD |
| 52 | 9 | M | CFPM, DOXY, GM, RFP | No | GC | 5.3 | PRD |
| 53 | 18 | F | DOXY, RFP | No | GC | 1.48 | PRD |
| 54 | 36 | M | GM, PCG, flucloxacillin | Yes | GC, CYC, AZA, MMF | 2.1 | CR |
| 55 | 78 | F | DOXY | No |  | 2.21 | PRD |
| 56 | 74 | M | DOXY, GM, macrolide | No |  | 2.43 | N/A |
| 57 | 74 | M | CAM, GM | No | GC, CYC | 5.5 | PRD |
| 58 | 59 | M | CTRX, DOXY, ST | Yes | GC | 5.5 | PRD |
| 59 | 64 | M | CTRX, DOXY, GM | Yes |  | 1.33 | CR |
| 60 | 72 | M | CTRX, DOXY, RFP | Yes | GC, CYC, MMF, HQ | N/A | N/A |
| 61 | 43 | M | CTRX, DOXY, GM, RFP | Yes | GC, CYC | N/A | N/A |
| 62 | 45 | M | CAZ | No |  | 3.8 | died |
| 63 | 36 | M | antibiotics | Yes | GC | 6.3 | CR |
| 63 | 76 | M | antibiotics | No | GC | 8 | died |
| 64 | 58 | M | DOXY, GM | Yes | GC, CYC | 4.57 | CR |
| 65 | 79 | M | DOXY, ofloxacin | No |  | 4.52 | N/A |
| 66 | 69 | M | CPFX, RFP | No |  | 3.39 | PRD |
| 67 | 53 | F | CTRX, DOXY | No | GC | 3.5 | died |
| 67 | 35 | M | DOXY, tobramycin | Yes | GC | 4.5 | PRD |
| 67 | 46 | M | AZM, CTRX | Yes |  | 5.2 | PRD |
| 68 | 53 | M | CAZ, ofloxacin | Yes |  | 2.05 | PRD |
|  |  |  |  |  |  |  |  |
| 69 | 74 | M | CTRX, DOXY | Yes | GC, HQ | 2.1 | N/A |
| 70 | 66 | M | DOXY, RFP | No |  | 2.6 | ESRD |
| 71 | 33 | M | CTRX, DOXY, GM | No |  | 3.3 | PRD |
| 72 | 44 | M | DOXY | Yes |  | >4 | ESRD |
| 73 | 62 | M | antibiotics | Yes |  | 2.61 | died |
| 74 | 71 | M | DOXY, GM | Yes | GC | N/A | PRD |
| 75 | 56 | M | DOXY, RFP | No | GC | 3.45 | CR |
| 75 | 42 | M | unknown | Yes |  | 4 | died |
| 75 | 64 | M | antibiotics | Yes | GC | 6 | PRD |
| 76 | 31 | F | DOXY, RFP | No | GC | N/A | PRD |
| 77 | 63 | M | DOXY | No |  | 3.8 | ESRD |
| 78 | 52 | M | unknown | Yes | GC, PLEX | 7 | N/A |
| 79 | 73 | M | antibiotics | Unknown |  | 5.9 | N/A |
| 80 | 79 | M | antibiotics | No |  | 2.14 | ESRD |
| 81 | 54 | M | antibiotics | Unknown |  | 2.5 | N/A |
| 82 | 56 | M | antibiotics | Yes |  | 3.2 | PRD |
| 83 | 55 | M | DOXY, RFP | No | GC | 5.5 | N/A |
| 84 | 20 | M | antibiotics | Yes |  | 3.1 | PRD |
| 85 | 37 | M | DOXY, RFP | Yes |  | 6.2 | PRD |
| 86 | 65 | M | unknown | No |  | 2.0 | died |
| Our | 74 | M | antibiotics, including CTRX | Yes |  | 2.5 | PRD |
| Our | 68 | M | DOXY | No |  | 3.46 | died |
| Our | 64 | M | DOXY | No | AZA | 5.8 | PRD |
| Our | 79 | M | DOXY, metronidazole | No |  | 4.2 | died |

N/A, not available; AMPC, amoxicillin, DOXY, doxycycline; GM, gentamycin; RFP, rifampicin; TAZ/PIPC, tazobactam piperacillin; CTRX, ceftriaxone; ST, Sulfamethoxazole and trimethoprim; MEPM, meropenem; CFPM, cefepime; PCG, penicillin G; CAM, clarithromycin; CAZ, ceftazidime; AZM, azithromycin; GC, glucocorticoid; CYC, cyclophosphamide; PLEX, plasma exchange; RTX, rituximab; MMF, mycophenolic mofetil; HQ, hydroxychloroquine; AZA, azathioprine; MPA, mycophenolate; Cr at bx, creatinine at biopsy; CR, complete recovery; PRD, persistent renal dysfunction; ESRD, end stage renal disease
